# Probable cerebral amyloid angiopathy diagnosed on plain CT

**DOI:** 10.1007/s10072-022-06330-2

**Published:** 2022-08-16

**Authors:** U. Jensen-Kondering, C. Flüh, C. Röcken, N. G. Margraf

**Affiliations:** 1 Department of Radiology and Neuroradiology, University Medical Center Schleswig-Holstein, Campus Kiel, Arnold-Heller-Str. 3, Haus D, 24105 Kiel, Germany; 2 Institute of Neuroradiology, University Medical Center Schleswig-Holstein, Campus Lübeck, Ratzeburger Allee 160, Haus A, 23538 Lübeck, Germany; 3 Department of Neurosurgery, University Medical Center Schleswig-Holstein, Campus Kiel, Arnold-Heller-Str. 3, Haus D, 24105 Kiel, Germany; 4 Department of Pathology, University Medical Center Schleswig-Holstein, Campus Kiel, Arnold-Heller-Str. 3, Haus U33, 24105 Kiel, Germany; 5 Department of Neurology, University Medical Center Schleswig-Holstein, Campus Kiel, Arnold-Heller-Str. 3, Haus D, 24105 Kiel, Germany

## Introduction

Cerebral amyloid angiopathy (CAA) is the most common cause for lobar haemorrhages. The prevalence of CAA is believed to be app. 30% in non-demented elderly patients and increases with age [1]. CAA is diagnosed using the modified Boston criteria [2] which relies heavily on neuroimaging.

Recently, the imaging signs “finger like projections” (FLP) and subarachnoid haemorrhage (SAH) adjacent to the main haemorrhage have been suggested as additional markers of CAA [3].

While MRI is desirable to advance the diagnosis of CAA non-invasively, in some patients, MRI is not feasible due to contraindications or logistics constraints. Moreover, plain CT remains the first-line imaging modality in the acute stage.

We present a retrospectively compiled case series of patients with lobar haemorrhages in whom an MRI was not performed but many imaging features hinted towards the diagnosis of cerebral amyloid angiopathy on plain CT.

## Case series

### Case 1

**Fig. 1:**
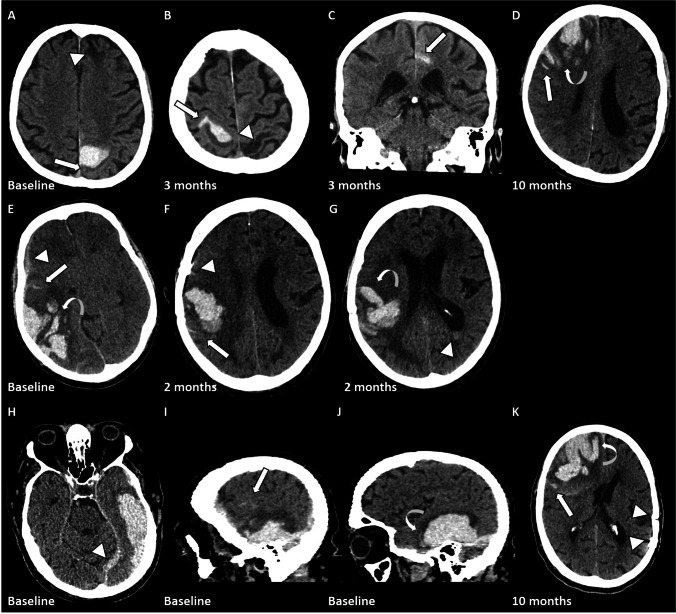
Case 1 (**A–D**), case 2 (**E**–**G**) and case 3 (**H**–**K**). **A** Left parietal lobar haemorrhage with adjacent SAH (straight arrow). The aetiology of the defect in the left frontal superior gyrus (triangle) remained elusive. **B** Lobar haemorrhage in the postcentral gyrus with adjacent SAH in the central sulcus (straight arrow). Note the left parietal defect (triangle). **C** Lobar haemorrhage in the left frontal superior gyrus and adjacent SAH (curved arrow) in the sulcus of the corpus callosum (coronal reconstruction). **D** Large right frontal lobar haemorrhage with mass effect, FLP (arrow) and SAH (curved arrow). FLP are extensions from the core haemorrhage which are longer than their width at the base of the haemorrhage. **E** Large right sided lobar haemorrhage with SAH (straight arrow), FLP (curved arrow) and subdural extension (triangle). **F** + **G** Large right sided lobar haemorrhage with SAH (**F**, straight arrow), FLP (**G**, curved arrow) and intraventricular extension (**G**, triangle). Note the trepanation (**F**, triangle). **H**–**J** Left temporal lobar haemorrhage with subdural (**A**, arrow) and subarachnoid extension (**I**, sagittal reconstruction, straight arrow) and FLP (**J**, sagittal reconstruction, curved arrow). **K** Large right frontal lobar haemorrhage with effect, SAH (straight arrow) and FLP (curved arrow). Note the trepanation (triangles)

An 81-year-old patient presented at a three months interval first with left and then with bilateral sensorimotor symptoms. CT demonstrates a left parietal lobar haemorrhage and bilateral frontal lobar haemorrhages with extension into the subarachnoid space involving the left sulcus of the corpus callosum and the right central sulcus (Fig. 1A-C). No surgical intervention was deemed necessary. Seven months after the second event, he presented with reduced level of consciousness. CT demonstrated right frontal lobar haemorrhage with SAH and FLP with mass effect (Fig. 1D). Craniotomy and hematoma evacuation were performed.

### Case 2

A 68-year-old male patient presented with reduced level of consciousness and severe left sided hemiparesis. Two months later, he again presented with reduced level of consciousness and severe left sided hemiparesis. On both instances, CT demonstrates a large right sided lobar haemorrhage with SAH and FLP (Fig. 1E-G). On both instances, craniotomy and hematoma evacuations were performed.

### Case 3

An 82-year-old male patient presented with headaches and severe non-fluent aphasia. CT demonstrated a left temporal lobar haemorrhage with subarachnoid and subdural extension. FLP could be demonstrated (Fig. 1H-J). Craniotomy and hematoma evacuations were performed. Ten months later, he presented with reduced level of consciousness and severe left sided hemiparesis. CT demonstrated a right frontal lobar haemorrhage with SAH and FLP (Fig. 1K). The patient deceased 1 day after admission.

**Fig. 2:**
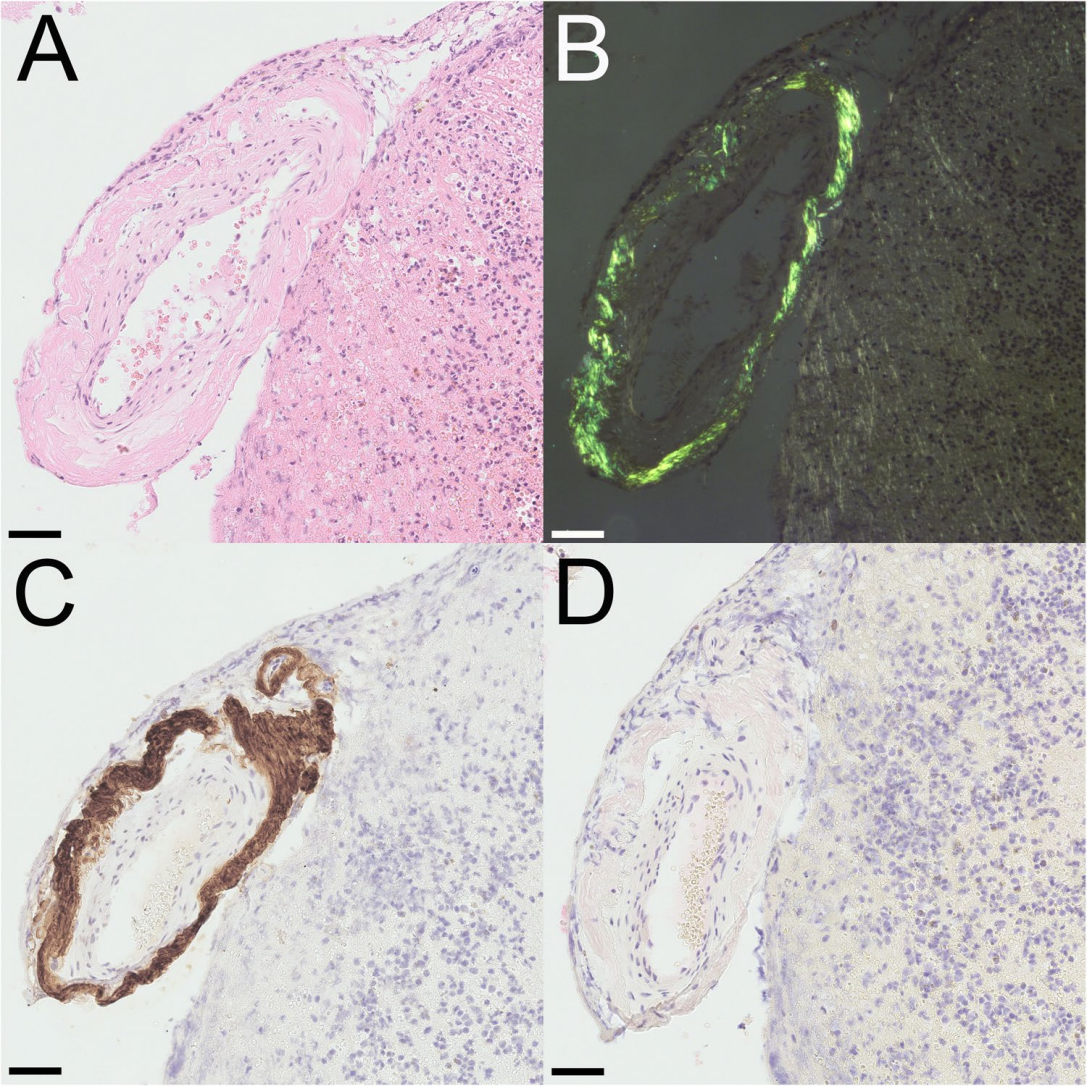
Tissue samples obtained during surgery were fixed in formalin and embedded in paraffin. Haematoxylin and eosin staining (**A**) showed thickened vessel walls. Following Congo red staining, a typical yellow-green birefringence was found in polarization microscopy (**B**). Immunostaining using antibodies directed against Aβ peptide classified the amyloid deposits as Aβ-amyloidangiopathy (**C**). No immunostaining of the vascular amyloid deposits was observed with an antibody directed against transthyretin (**D**). Scale bar: 25 µm

In all cases, specimens obtained from hematoma evacuation underwent neuropathological assessment including immunohistochemistry staining which demonstrated severe Aβ-amyloid angiopathy (Fig. 2).

## Discussion

The modified Boston criteria require evidence for multiple haemorrhagic lesions demonstrated either clinically, on CT or MR for the diagnostic category “probable cerebral amyloid angiopathy” [2]. The cases presented here have in common multiple haemorrhagic lesion or residuals thereof in lobar location demonstrated on plain CT with clinical sequela. In all cases, immunohistology confirmed Aβ-amyloid angiopathy thereby elevating diagnostic certainty of these lesions. Additionally, all haemorrhages demonstrated adjacent subarachnoid haemorrhage and almost all haemorrhages demonstrated finger-like projections, both being potential imaging markers of CAA [3].

These cases remind us that plain CT holds a wealth of information if MRI is not available.
